# Urethral Hemangioma Treated With Transurethral Coagulation Using Narrow-Band Imaging: A Case Report

**DOI:** 10.7759/cureus.82791

**Published:** 2025-04-22

**Authors:** Junichi Ikeda, Hisanori Taniguchi, Monta Inoue, Yuki Masuo, Takahiro Nakamoto, Katsunori Uchida, Masaaki Yanishi, Hidefumi Kinoshita

**Affiliations:** 1 Urology and Andrology, Kansai Medical University, Hirakata, JPN; 2 Pathology, Kansai Medical University, Hirakata, JPN

**Keywords:** artificial erection, gross hematuria, narrow-band imaging (nbi), transurethral coagulation, urethral hemangioma

## Abstract

A 49-year-old man consulted his previous doctor, reporting occasional episodes of macroscopic hematuria after an erection. Cystourethroscopy under artificial erection revealed findings suggestive of a hemangioma in the membranous urethra. The frequency of hematuria decreased with the use of 5α-reductase inhibitors (5ARIs) and hemostatic agents but subsequently increased, leading to the patient’s referral to our department for further examination and treatment. Under general anesthesia and in the lithotomy position, an artificial erection was induced by injecting 80 mg of papaverine hydrochloride into the corpus cavernosum, and a cystourethroscopy was performed using white light. A mass was identified extending from the distal verumontanum of the prostatic urethra to the membranous urethra. Due to the proximity of the hemangioma to the urethral sphincter, narrow-band imaging (NBI) was used for precise identification, followed by biopsy and coagulation. The histopathological findings were a cavernous hemangioma. The patient experienced no recurrence of hematuria or urinary incontinence. Urethral hemangioma, a known cause of posterectile hematuria, can be effectively diagnosed and treated via endoscopic observation during induced erection. In this case, NBI facilitated the accurate visualization of the hemangioma, enabling surgical resection without postoperative complications such as urinary incontinence, despite the mass’s proximity to the urethral sphincter. NBI improves the visibility of the mass and may contribute to more accurate and safer treatment of urethral hemangiomas.

## Introduction

Urethral hemangioma is a relatively rare and benign hemangioma originating within the urethra [1]. Major symptoms of urethral hemangioma include hematuria, urethral bleeding, and urinary retention; however, they may also be asymptomatic [2]. Although cystourethroscopy is considered useful for diagnosis [3], some cases are only observable during penile erection. Therefore, if diagnosis cannot be established through conventional cystourethroscopy, inducing an artificial erection is required [4]. In this report, we describe a case in which transurethral coagulation of a hemangioma near the urethral sphincter was performed safely using narrow-band imaging (NBI) during induced erection.

## Case presentation

A 49-year-old married male patient with children occasionally presented with macroscopic hematuria occurring after erection. His past medical history included hypertension, and he was prescribed dutasteride and olopatadine. Five years prior, he had visited a physician with the same complaint. Cystourethroscopy under artificial erection revealed a suspected hemangioma near the membranous urethra. Due to concerns about the risk of urinary incontinence following transurethral surgery, a conservative management approach using a 5α-reductase inhibitor (5ARI) and hemostatic agents was chosen. Despite an initial decrease in the frequency of hematuria, complete resolution was not achieved, and the symptoms gradually worsened. Consequently, he visited our department in year X for further evaluation and treatment. Laboratory findings are shown in Table 1.

**Table 1:** Laboratory findings of the patient NGSP: National Glycohemoglobin Standardization Program

Laboratory test	Result	Normal range
Red blood cell count (×10⁶/μL)	4.87	4.0–5.7
Hemoglobin (g/dL)	13.1	12.9–17.2
Hematocrit (%)	40.7	38.2–50.8
White blood cell count (/μL)	5,200	3,500–8,500
Platelet count (×10³/μL)	276	140–340
Creatinine (mg/dL)	0.76	0.6–1.0
C-reactive protein (mg/dL)	0.01	<0.3
HbA1c (NGSP) (%)	5.5	4.6–6.2
Activated partial thromboplastin time (sec)	26.7	24–34
Prothrombin time-international normalized ratio	0.95	Unknown
Prostate-specific antigen (ng/mL)	0.5	<4.0

Urinalysis revealed no proteinuria, glycosuria, or hematuria, with red blood cell (RBC) 0-1/HPF, white blood cell (WBC) 0-1/HPF, no epithelial cells, and no bacteria detected. Urine cytology was negative. Contrast-enhanced CT revealed no apparent mass lesions in the urethra.

With the patient’s consent, we performed transurethral resection or coagulation of the urethral hemangioma under general anesthesia. To ensure accurate identification of the tumor and avoid damage to the sphincter, we used NBI.

**Figure 1:**
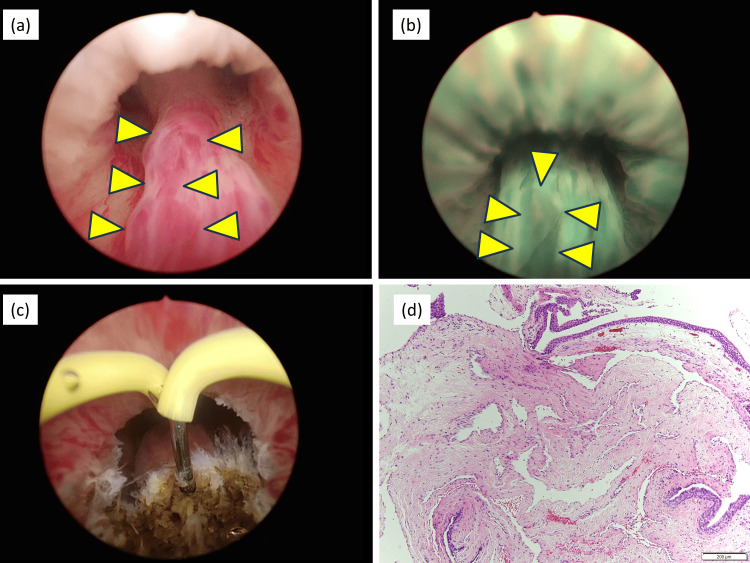
Cystourethroscopy and pathology images (a) Urethral hemangioma observed after induction of artificial erection. (b) Urethral hemangioma observed under narrow-band imaging. (c) Hemangioma after transurethral electrocoagulation with a needle electrode. (d) Histopathological examination of hematoxylin and eosin stain showing a cavernous hemangioma with dilated vessels within the stromal region.

In year X, under general anesthesia and in the lithotomy position, an artificial erection was induced by locally injecting 80 mg of papaverine hydrochloride into both corpora cavernosa. Cystourethroscopy under white light revealed a tumor extending from the distal verumontanum of the prostatic urethra to the membranous urethra (Figure 1a).

The surface of the tumor appeared red under white light, while NBI clearly delineated the lesion as a vascular structure (Figure 1b). A biopsy of the tumor was performed, followed by coagulation with a needle electrode (Figure 1c). The patient was discharged on postoperative day 3 without complications.

Histopathological examination confirmed a cavernous hemangioma, characterized by dilated vascular structures within the stromal region (Figure 1d). At the three-month follow-up, the patient exhibited no recurrence of hematuria, urinary incontinence, or hematospermia.

## Discussion

Urethral hemangioma is a relatively rare benign vascular tumor that has been reported as a cause of hematuria. Including the present case, a total of 60 cases of urethral hemangioma have been reported in men (Table 2) [1-31].

**Table 2:** Clinical features of male urethral hemangioma KTP: potassium titanyl phosphate; Nd-YAG: neodymium-doped yttrium aluminum garnet; Ho-YAG: holmium-doped yttrium aluminum garnet

Case	Authors	Published year	Age	Symptom	Causes, triggers of symptom onset	Size/location of hemangioma	Treatment
1	Cattolica [4]	1982	31	Hematospermia, hematuria	Unknown	Unknown	Fulguration
2	Cattolica [4]	1982	32	Hematospermia, hematuria	Unknown	Unknown	Fulguration
3	Cattolica [4]	1982	NA	Hematospermia, hematuria	Unknown	Unknown	Fulguration
4	Redman and Young [5]	1987	29	Hematospermia, hematuria	Unknown	Unknown	Fulguration
5	Borrego Hernando and Maganto Pavón[6]	1996	45	Urethrorrhagia	Following normal erection	Unknown	Argon laser
6	Lauvetz et al. [7]	1996	7	Hematuria	Unknown	-/anterior urethra	KTP/532 laser
7	Hayashi et al. [8]	1997	30	Hematospermia, hematuria	Unknown	5 mm/between the verumontanum and external sphincter	Resection
8	Furuya et al. [9]	1997	53	Hematospermia, hematuria	Postejaculation	5 × 4 × 3 mm/distal to the vermontanum	Resection
9	Khaitan and Hemal [10]	2000	14	Hematuria	Unknown	1-5 mm/penile urethra	Nd-YAG laser
10	Parshad et al. [11]	2001	23	Hematuria	Unknown	10 × 10 mm/Bullbar urethra	Vertical penoscrotal wide excision
11	Wilson et al. [12]	2001	36	Urethral bleeding	Blue rubber bleb nevus syndrome	-/anterior urethra	Conservative
12	Demircan et al. [13]	2006	18	Hematuria	Unknown	22 × 9 mm/bladder neck	Excision by cystostomy
13	Terada et al. [14]	2007	24	Urethral bleeding	Klippel-Trenaunay-Weber syndrome	-/anterior urethra	Endoscopic sclerotherapy by injecting 5% solution of monoethanolamine oleate (Oldamine)
14	de León et al. [15]	2008	31	Hematuria, clot expulsion, urinary retention	Unknown	-/prostatic urethra	Ho-YAG laser
15	Saito [1]	2008	50	Hematospermia, hematuria	Unknown	Unknown	Resection
16	Saito [1]	2008	70	Hematospermia, hematuria	Unknown	Unknown	Resection
17	Saito [1]	2008	38	Hematospermia, hematuria	Unknown	Unknown	Resection
18	Saito [1]	2008	41	Hematospermia, hematuria	Unknown	Unknown	Resection
19	Saito [1]	2008	67	Hematospermia, hematuria	Unknown	Unknown	Resection
20	Saito [1]	2008	51	Hematospermia, hematuria	Unknown	Unknown	Resection
21	Saito [1]	2008	80	Hematuria	Unknown	Unknown	None
22	Saito [1]	2008	68	Hematuria	Unknown	Unknown	Resection
23	Saito [1]	2008	75	Hematuria	Unknown	Unknown	Resection
24	Saito [1]	2008	55	Hematuria	Unknown	Unknown	Resection
25	Saito [1]	2008	63	Hematuria	Unknown	Unknown	Resection
26	Saito [1]	2008	67	None	Unknown	Unknown	Resection
27	Saito [1]	2008	56	None	Unknown	Unknown	Resection
28	Saito [1]	2008	67	None	Unknown	Unknown	Resection
29	Saito [1]	2008	58	None	Unknown	Unknown	Resection
30	Saito [1]	2008	56	None	Unknown	Unknown	Resection
31	Saito [1]	2008	67	None	Unknown	Unknown	Resection
32	Saito [1]	2008	69	None	Unknown	Unknown	Resection
33	Saito [1]	2008	77	None	Unknown	Unknown	Resection
34	Saito [1]	2008	82	None	Unknown	Unknown	Resection
35	Efthimiou et al. [3]	2009	27	Urethral bleeding	Unknown	5 mm/anterior urethra	Excision by biopsy forceps
36	Noviello et al. [16]	2011	1	Hematuria	Unknown	-	Transurethral excision
37	Tepeler et al. [17]	2011	8	Urethral bleeding	Klippel-Trenaunay syndrome	-/anterior urethra	Conservative
38	Abbinante et al. [18]	2012	18	Urethral bleeding	Unknown	-/6 cm far from the external urethral meatus	Surgical removing
39	Singh and Mandal [19]	2013	14	Urethral bleeding	Unknown	5 mm/pendular urethra	Transurethral ablation using Ho-YAG laser
40	Han et al. [20]	2015	54	Hematuria	After ejaculation	-/prostatic urethra	Transurethral resection
41	Han et al. [20]	2015	39	Hematuria	After ejaculation	-/prostatic urethra	Transurethral resection
42	Han et al. [20]	2015	55	Hematuria	After ejaculation	-/prostatic urethra	Transurethral resection
43	Han et al. [20]	2015	44	Hematuria	After ejaculation	-/prostatic urethra	Transurethral coagulation
44	Han et al. [20]	2015	39	Hematuria	After ejaculation	-/prostatic urethra	Transurethral resection
45	Hamada et al. [21]	2017	73	Hematuria, dysuria	After ejaculation	-/prostatic urethra	Transurethral resection
46	Soleimani et al. [22]	2017	41	Hematuria, urethral bleeding	Unknown	5 mm/anterior urethra	Monopolar electrocautery, Coagulated with Ho-YAG laser
47	Soleimani et al. [22]	2017	22	Hematuria	Postejaculatory hematuria	10 mm/12 o’clock of anterior urethra	Coagulated with Ho-YAG laser
48	Soleimani et al. [22]	2017	14	Urethral bleeding	Unknown	10 mm, 15 mm/4 o’clock of anterior urethra	Coagulation by Ho-YAG laser
49	Itesako et al. [23]	2018	3	Urethral bleeding	Unknown	-/bulbar urethra	Oral propranolol
50	Varea-Malo et al. [24]	2019	61	Urethral bleeding	Unknown	70 mm/anterior urethra and prostatic urethra with bulbar stricture	Conservative
51	Yong et al. [25]	2019	15	Hematuria	Unknown	-/membranous urethra	Injection of pingyangmycin
52	Yong et al. [25]	2019	49	Hematuria, urethral bleeding	After penile erection	1.1 × 2.4 cm/distance of 2.4 cm from urethral meatus	Injection of pingyangmycin
53	Masood et al. [26]	2021	18	Urethral bleeding	Unknown	-/5 o’clock of posterior urethra	Fulguration by diathermy and intralesional triamcinolone
54	Carolan et al. [27]	2022	6	Hematuria	Unknown	8 × 8 × 6 mm/prostatic urethra	Transurethral resection
55	Genov et al. [28]	2022	64	Urethral bleeding	Unknown	7 mm/navicular fossa	Coagulation by Thulium YAG laser
56	Qian et al. [29]	2022	36	Hematuria	Postejaculatory hematuria	-/5 o’clock of posterior urethra	Excision by plasma electrodes
57	Ishikawa et al. [2]	2023	45	Painless hematuria, blood clots, urinary retention	After erection/ejection	6 × 6 mm/prostatic urethra	Transurethral resection
58	Pal et al. [30]	2023	14	Hematuria	Unknown	Urethra	Coagulation by Ho-YAG laser
59	Alfentoukh et al. [31]	2023	35	Urethral bleeding	Unknown	10 mm/verumontanum	Resection
Our case	Ikeda et al.	2025	49	Hematuria	After erection	-/from the distal verumontanum of the prostatic urethra to the membranous urethra	Transurethral coagulation

The median age of the patients was 41 years (range: 1-77 years), and 51 of the 60 cases presented with hematuria or urethral bleeding. Among the 38 cases of hematuria, 12 were associated with hematospermia. Postejaculatory hematuria was reported in 10 cases, whereas four cases (including one case with postejaculatory hematuria) experienced hematuria after erection. Hemangioma locations varied, with the prostatic urethra being the most frequently reported site (14 cases). Regarding hemostatic treatment, transurethral resection was the most commonly performed procedure, reported in 34 cases. Laser therapy was performed in 10 of the 60 cases.

The exact cause of urethral hemangioma remains unclear; congenital and acquired theories have been proposed. The congenital theory suggests that it may result from the fetal remnants of angioblastic cells [8], whereas the acquired theory attributes it to repeated increases in venous pressure [1]. According to a report by Saito [1], increased intraurethral pressure during erection or ejaculation may cause vascular rupture, leading to bleeding. Additionally, cases associated with congenital disorders such as Klippel-Trenaunay [14,17] and blue rubber bleb nevus syndromes [12] have also been reported.

Symptoms of urethral hemangiomas vary depending on their location. Cases with hemangiomas in the distal urethra are more likely to present with urethral bleeding, whereas those with hemangiomas near the proximal urethra tend to develop hematuria or urinary retention [26]. In addition, larger hemangiomas have been reported to cause urinary retention or protrusion of the hemangioma from the external urethral meatus [26].

Cystourethroscopy, typically performed in an outpatient setting under local anesthesia, remains the standard method for identifying hemangiomas; however, some cases have been reported in which the lesion could only be detected during erection. Performing cystourethroscopy after inducing an artificial erection has been suggested to improve the visualization of hemangiomas [4]. Additionally, penile ultrasonography and transrectal power Doppler ultrasonography have been reported as useful modalities for detecting enlarged soft tissue and increased blood flow [1]. NBI is a technique that enhances the visualization of blood vessels by improving image resolution and contrast [32]. In the present case, the hemangioma was identifiable under white light after inducing an artificial erection. However, observation under NBI allowed for a more accurate identification of the lesion.

The management of urethral hemangioma depends on the presence of symptoms. Asymptomatic cases can be observed without intervention, but symptomatic cases require some form of treatment, such as coagulation or resection [3]. In recent years, the use of laser coagulation for treatment has been reported [11]. Although 5ARIs have been reported to reduce perioperative bleeding during transurethral resection of the prostate [33], their efficacies in preventing spontaneous bleeding from urethral hemangiomas have not been reported. In addition, 5ARIs have been associated with an increased risk of depression [34], making long-term use difficult in this case.

In this case, we decided to perform transurethral coagulation using endoscopy. Since the hemangioma was located near the urethral sphincter, we aimed to avoid sphincter damage from a resectoscope electrode. By accurately identifying the lesion with NBI, we performed coagulation using a needle electrode and successfully completed the procedure without postoperative complications. To the best of our knowledge, this is the first report of transurethral coagulation of hemangioma using NBI.

## Conclusions

This study demonstrates that NBI allowed accurate visualization of the hemangioma, enabling surgical resection without postoperative complications. This suggests that NBI improves the visibility of urethral hemangiomas. Therefore, NBI may contribute to more accurate and safer treatment of these lesions.
